# Restraint theory: Significance of rumination

**DOI:** 10.1192/j.eurpsy.2021.476

**Published:** 2021-08-13

**Authors:** A. Brytek-Matera, P. Bronowicka, J. Walilko

**Affiliations:** 1 Katowice Faculty Of Psychology, SWPS University of Social Sciences and Humanities, Katowice, Poland; 2 Institute Of Psychology, University of Wroclaw, Wroclaw, Poland

**Keywords:** dieting, uncontrolled eating, emotional eating, restraint theory

## Abstract

**Introduction:**

Restraint theory (Herman and Polivy, 1975) suggests that human eating behaviour is under cognitive control and this leads to reduced sensitivity to internal cues for satiety, resulting in overeating in situations where cognitive control is under-mined (Johnson et al., 2012). In other words, restraint theory suggests that restraint (dieting) actually leads to leads to an excessive intake of food.

**Objectives:**

The present study sought to investigate the relationship between dieting, eating behaviours (uncontrolled eating, emotional eating, cognitive restraint) and rumination (repetitive negative thinking). The second objective was to determine whether rumination mediates the relationship between dieting and both uncontrolled eating and emotional eating.

**Methods:**

The sample was composed of 188 women (M_age_ = 29.46 ± 8.94; M_BMI_ = 23.16 ± 4.04). The Eating Attitudes Test, the Three-Factor Eating Questionnaire and the Perseverative Thinking Questionnaire were used in the present study.

**Results:**

Dieting for weight control (intentional weight loss) was associated with higher levels of uncontrolled eating, emotional eating, cognitive restraint and repetitive negative thinking. Mediation analyses showed that the relationship between dieting and inappropriate eating behaviours was mediated by rumination. The direct effect of dieting on both uncontrolled eating and emotional eating was significant, suggesting partial mediation.

**Conclusions:**

Our findings support the relevance of rumination in linking dieting and eating behaviours among women. The current study may have clinical applications such as the potential integration of rumination for the prevention and changes in inappropriate eating behaviours.

**Disclosure:**

No significant relationships.

